# Comprehensive review on molnupiravir in COVID-19: a novel promising antiviral to combat the pandemic

**DOI:** 10.2217/fmb-2021-0252

**Published:** 2022-02-24

**Authors:** Sajad Khiali, Elnaz Khani, Samineh B Rouy, Taher Entezari-Maleki

**Affiliations:** 1 Department of clinical pharmacy, Faculty of pharmacy, Tabriz University of Medical Sciences, Tabriz, 5166414766, Iran; 2 Student Research Committee, Tabriz University of Medical Sciences, Tabriz, Iran; 3 Cardiovascular research center, Tabriz University of Medical Sciences, Tabriz, 5166615573, Iran

**Keywords:** COVID-19, direct-acting antivirals, molnupiravir, MK-4482, SARS-CoV-2

## Abstract

Despite the progress in the management of COVID-19, effective oral antiviral agents are still lacking. In the present review, the potential beneficial effects of molnupiravir in the management of COVID-19 are discussed. A literature search in Google Scholar, Scopus, PubMed and clinicaltrials.gov for the relevant articles regarding the pharmacokinetics, pharmacodynamics and clinical trials of molnupiravir in the management of COVID-19 is conducted. Most of the preclinical studies and available clinical trials showed a favorable short-term safety profile of molnupiravir; however, given its possible genotoxic effects, further trials are required to confirm the long-term efficacy and safety of molnupiravir in patients with COVID-19.

The world has been in pandemic mode since the WHO's declaration of COVID-19 as a pandemic on 11 March 2020. At the time of writing the manuscript, SARS-CoV-2, the causal agent of COVID-19, has infected more than 331 million patients with approximately 5.55 million deaths globally [1].

The clinical disease manifestation ranges from asymptomatic cases to inflammatory complications, acute respiratory distress syndrome, thromboembolic events and multi-organ damage. Despite considerable advances in the prevention and treatment of SARS-CoV-2 infection, the pandemic has not been controlled appropriately [1–3].

As of 19 January 2022, more than 60% of the globe population have received at least one dose of COVID-19 vaccine, and about 9.7 billion vaccine doses have been administered globally, with a sharp gap between vaccination programs in different countries. Notably, the efficacy and safety of the vaccines in real-world conditions especially in long-term and against new variants of the SARS-CoV-2, such as Omicron variant, have not been fully understood. Furthermore, the vaccine does not completely prevent individuals from infection by SARS-COV-2, and new cases of COVID-19 have been reported following a full shot of the vaccine. Finally, there are substantial concerns about vaccine development duration and cost. Altogether, there is an urgent need for effective antiviral drugs to combat the disease and control the pandemic [4–6].

According to the NIH guidelines, the main therapeutic candidates for patients with COVID-19 include antiviral drugs, cell-based therapy, anti-SARS-CoV-2 antibody products, corticosteroids and immunomodulators, antithrombotic therapy and supplements [7]. Among these therapeutic options, antivirals have attracted the greatest attention because of their key role in the prevention of viral transmission to high-risk individuals and treating infected patients with COVID-19, particularly in the early stages of the diseases [7]. Currently, remdesivir, a ribonucleic acid (RNA)-dependent RNA polymerase (RdRp) inhibitor, is the only antiviral agent that has been approved by the United States Food and Drug Administration (FDA) for the treatment of COVID-19; however, randomized clinical trials have produced conflicting results about the efficacy of remdesivir. A systematic review and meta-analysis of randomized controlled trials showed that remdesivir could significantly reduce the mortality rate (RR = 1.09, 95%CI, 1.04–1.15) [8]. Notably, the NIH panel opposed the routine administration of remdesivir in individuals on mechanical ventilation because of the lack of data regarding the benefits of the drug at the advanced stage of the disease. Furthermore, remdesivir administration in a hospital or a health care setting with a comparable level of care to a hospital could limit the wide use of the drug in the early stages of the disease [8].

It seems that vaccination and antiviral treatment are the two fundamental tools to stop the pandemic and treat patients with severe COVID-19. Because of the increasing demand for treatment strategies in life-threatening conditions like the COVID-19 pandemic, drug repurposing approach and off-label treatment administration are unavoidable. Today our knowledge about the pathogenesis of COVID-19 advanced substantially, and more specific, safe and effective antiviral drugs repurposing and development against the virus is important [2,9].

Several medications, such as Paxlovid™ (PF-07321332; ritonavir), umifenovir, emetine, brilacidin, stannous protoporphyrin and molnupiravir have been launched or are in clinical trials for the management of COVID-19. Molnupiravir is an orally bioavailable ribonucleoside analog with promising results in the management of COVID-19 disease [10]. It has been shown that molnupiravir is efficacious against remdesivir-resistant SARS-CoV-2 and multiple distinct SARS- or MERS-like viruses (bat coronavirus: SHC014, HKU3 and HKU5). These data suggest that mutant variants of SARS-CoV-2 infection and even outbreaks of future zoonotic coronaviruses would be susceptible to molnupiravir [11].

Given these points, we aimed to review the current evidence regarding pharmacodynamic, pharmacokinetic and clinical trials in patients with COVID-19.

## Methods

A literature search in Google Scholar, Scopus, PubMed and clinicaltrials.gov for the relevant articles regarding the pharmacokinetics, pharmacodynamics and clinical trials of molnupiravir in the management of COVID-19 from database inception to 20 January 2022 was conducted. Our search terms were (‘antiviral’ OR ‘NHC’ OR ‘EIDD-1931’ OR ‘molnupiravir’ OR ‘MK-4482’ OR ‘EIDD-2801’ OR ‘pathogenesis’) AND (‘COVID-19’ OR ‘SARS-CoV-2’ OR ‘2019 novel coronavirus’ OR ‘2019-nCov’). Duplicate publications, reviews, commentaries, case reports were excluded from the study. The related articles to our review have been discussed.

## Virology & pathogenesis

The Coronaviridae family is divided into two subfamilies of the Coronavirinae and the Torovirinae. The subfamily Coronavirinae is further divided into alpha, beta, gamma and delta coronavirus genera. The human coronaviruses are in alpha (229E and NL63) and beta (OC43, HKU1, MERS-CoV, SARS-CoV and SARS-CoV-2) genera [12]. Similar to other coronaviruses, SARS-CoV-2 is an enveloped virus with a positive-sense single-stranded RNA genome. The SARS-CoV-2 genome length is approximately 30 kb with 14 open reading frames, encoding at least 27 structural, non-structural and accessory proteins [13].

The replication of SARS-CoV-2 depends on a sequence of steps. Cell entry of SARS-CoV-2 principally depends on binding of the receptor-binding domain of viral S protein to the ACE2 protein located at the host cell surface and on S protein priming via the cellular transmembrane serine protease (TMPRSS2). Furthermore, it has been shown that the endosomal/lysosomal cysteine proteases cathepsin B and L probably play a role in the cell entry of the virus [14,15].

Following virus entry into the cytoplasm of the host cells, the uncoated genomic RNA is translated to produce two polyproteins (pp1a/ab) from two open reading frames and subsequently assembled into replication/transcription complexes (RTCs) with virus-induced double-membrane vesicles (DMVs). Afterward, the RTC replicates continuously and synthesizes a nested set of subgenomic RNAs by genome transcription, encoding proteins. Finally, the produced virus particles assembly, budding and exocytosis into the extracellular milieu compartment occur through the host cell components, such as the endoplasmic reticulum and Golgi complex. Therefore, the viral replication cycle and progression initiate, leading to host cell damage, immune system dysregulation, hypercoagulable state and multi-organ damage [16,17].

Antiviral agents could combat COVID-19 through multiple mechanisms, such as viral entry blocking, targeting viral enzymes, blocking the formation of virus particles, and inhibiting host factors required for viral replication. The steps in the viral replication process of SARS-CoV-2 are potential targets for antiviral agents. For example, remdesivir, the first FDA approved agent against COVID-19, targets the RdRp to prevent the viral RNA synthesis and has clinical benefits in patients with COVID-19 [18,19].

The RdRp plays a leading role in the replication and transcription of viral RNA. It has been shown that nsp12 has considerable polymerase activities along with nsp7 and nsp8, whereas nsp12 itself has limited catalytic activity. The complex of SARS-CoV-2 RdRp contains nsp12 core catalytic unit, nsp7, nsp8-1 and nsp8-2 [20,21].

Owing to the key role of RdRp in the replication of the RNA genome as well as the nonexistence of a counterpart to RdRp in mammalian cells, it has been a key therapeutic target in drug discovery and development against SARS-CoV-2 [20,21].

## Molnupiravir pharmacodynamics

### Mechanism of action

Numerous RdRp inhibitors have been launched or are under investigation in clinical trials for the management of COVID-19. Nucleoside analog inhibitors and nonnucleoside analog inhibitors, two well-known classes of RdRp inhibitors, bind to the enzyme at the enzyme active and allosteric sites, respectively. RdRp nucleoside inhibitors are categorized into purine, pyrimidine and miscellaneous subtypes [10].

**Figure 1.**
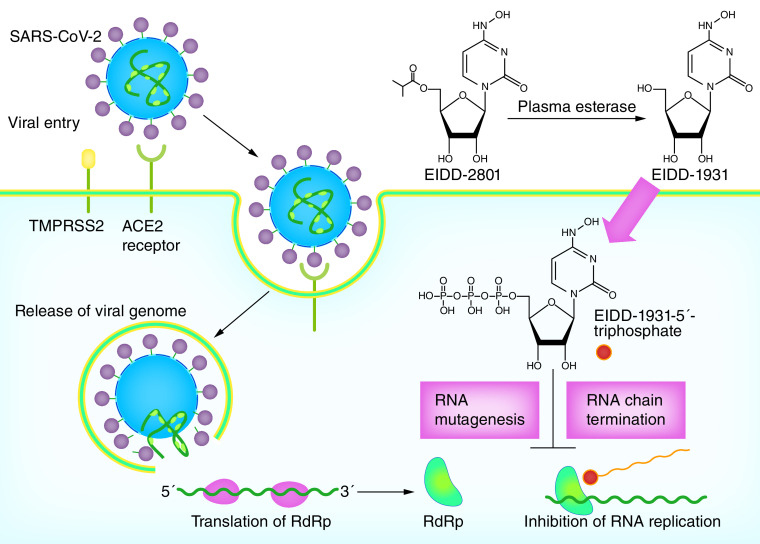
The mechanisms of action of molnupiravir against SARS-CoV-2. Molnupiravir (EIDD-2801) is converted to its active form NHC (EIDD-1931) via plasma esterase. NHC is distributed throughout the fluid and tissues in the body. In the host cells, NHC is phosphorylated to form NHC 5′-triphosphate. The RdRp wrongly uses NHC 5′-triphosphate instead of uridine triphosphate and cytidine triphosphate, which resulted in RdRp inhibition, RNA chain termination and mutagenesis. ACE-2: Angiotensin convertin enzyme 2.

The orally available 5′-isobutyric ester form of ribonucleoside analog ß-d-N4-hydroxycytidine (NHC or EIDD-1931), molnupiravir (MK-4482 [known previously as EIDD-2801]; Merck) is an oral ribonucleoside analog with promising results in the management of COVID-19. Molnupiravir is immediately converted to the active antiviral NHC by plasma esterase (Figure 1). NHC is distributed into different tissues and undergoes to intracellular phosphorylation via host cell kinase to form NHC 5′-triphosphate, which is an alternative substrate for the viral RdRp. It has been shown that molnupiravir functions as an RNA mutagen rather than a chain terminator, which resulted in lethal transition mutations, accumulate in viral RNA and viral extinction [10,22–26]. It has been indicated that the treatment with NHC is associated with increased mutation rate in viral genomic RNA of influenza, Venezuelan equine encephalitis virus (VEEV) and Rous sarcoma virus [26,27]. Sheahan *et al.* showed that either remdesivir or NHC could decrease virus titers dose-dependently in human primary airway epithelial cell cultures; however, the error frequency in remdesivir-treated group was low. While, the error frequency of MERS-CoV RNA was significantly higher in the NHC group at 24 (10 μM; p < 0.0001, 1 μM; p < 0.0001) and 48 (10 μM; p < 0.0001; 1 μM; p = 0.0015) h post-infection in a dose-dependent manner. Accordingly, the treatments with 1 μM NHC and 10 μM NHC led to a threefold (138-fold decrease in virus titer) and a sixfold (26,000-fold decrease in virus titer) increase in error rate, respectively. Furthermore, data showed that treatment with NHC is associated with adenine-to-guanine and uracil-to-cytosine transitions in MERS-CoV RNA [24]. Similarly, in Rosenke *et al.* study in the Syrian hamster model infected with SARS-CoV-2, evaluation of viral RNA isolated from lung samples showed that molnupiravir as a cytosine analog causes increased adenosine-to-guanosine and cytosine-to-uracil transitions in viral genomes [28].

### Preclinical studies

#### Antiviral activity

A mounting body of evidence has demonstrated the activity of NHC against several RNA viruses in infected cell culture models [29–35]. For example, Painter *et al.*, in a murine model of lethal VEEV infection, showed that the administration of ribonucleoside analog NHC within 24 h post-infection is 90–100% effective against the virus. Furthermore, it showed good tolerability after 7-day repeated dosing (up to 1000 mg/kg/day) [36].

It has been shown that the active agent of molnupiravir has antiviral properties against several RNA viruses, such as VEEV, Eastern and Western EEV, chikungunya virus, mouse hepatitis virus, highly pathogenic influenza viruses, and three known zoonotic coronaviruses, including MERS-CoV, SARS-CoV and SARS-CoV-2 [25,27,36]. Notably, Sheahan *et al.* showed that molnupiravir was effective against remdesivir-resistant SARS-CoV-2 as well as multiple genetically distinct bat-CoV, including SARS-like (HKU3 and SHC014) and MERS-like (HKU5) viruses [24].

In Sheahan *et al.* study [24], assessment of the antiviral activity of NHC against MERS-CoV in the human lung epithelial cell line Calu-3 2B4 (Calu-3 cells) revealed that it is a potent antiviral (half-maximal inhibitory concentration (IC_50_) = 0.15 μM). NHC was also found to be a potent antiviral against SARS-CoV-2 (2019-nCoV/USA-WA1/2020) in African green monkey kidney (Vero) cells model (IC_50_ = 0.3 μM, CC_50_ >10 μM). Also, evaluation of the antiviral effects of NHC against SARS-CoV-2 showed a dose-dependent reduction in virus titers (IC_50_ = 0.08 μM) and viral genomic RNA (IC_50_ = 0.09 μM) in the Calu-3 cells. Furthermore, a series of investigations in primary human airway epithelial cell cultures revealed that NHC could block SARS-CoV-2 infectious virus production in a dose-dependent manner. Besides, it could reduce virus production in MERS-CoV and SARS-CoV-infected human airway epithelial cell cultures with averages IC_50_ of 0.024 μM and 0.14 μM, respectively.

Following the promising *in vitro* antiviral effects of NHC against these coronaviruses, a prophylactic dose-escalation study was carried out in C57BL/6 mice. The mice were given vehicle or molnupiravir (50, 150, or 500 mg/kg) every 12 h, starting two h before intranasal infection with SARS-CoV. In comparison to the control group, molnupiravir could diminish or prevent weight loss (all ps < 0.0001). Moreover, there was a significant reduction in lung hemorrhage in mice groups treated with molnupiravir 500 mg/kg (p = 0.010). Finally, treatment with molnupiravir with doses of 150 and 500 mg/kg resulted in a significant dose-dependent reduction in the lung titer of SARS-CoV (ps = 0.03 and 0.006, respectively). Similar results were also observed in mice infected MERS-CoV samples regarding the reduction of viral load (all ps < 0.0001), body weight loss (ps = 0.03 to <0.0001) and lung hemorrhage (ps = 0.01 to <0.0001).

Given the benefits of 500 mg/kg molnupiravir in the prophylactic dose-escalation study, the effects of 500 mg/kg molnupiravir were evaluated under therapeutic conditions (starting 12, 24, or 48 h after infection). In the prophylactic phase, molnupiravir was administrated two h prior to exposure with SARS-CoV. All groups were given molnupiravir every 12 h during the study period. Data analysis showed that both prophylactic and therapeutic doses of the drug significantly impeded weight loss two days after infection and later, compared to the vehicle group (2 h before infection: ps = 0.0002 to <0.0001; 12 h after infection: ps = 0.0289 to <0.0001); while starting treatment 24 and 48 h after the infection led to a significant loss of body weight from the third (ps = 0.01 to <0.0001) and fourth day (p = 0.037) after infection, respectively. Interestingly, the viral load was lower in all molnupiravir-treated groups regardless of the time of starting the drug (all ps < 0.0001). The results also showed that the lung hemorrhage was significantly lower when the treatment was initiated during the first 24 h of infection (all ps < 0.0001). Improvement in pulmonary function was achieved when the drug was started 2 h before or 12 h after infection (ps < 0.0001 to 0.019 and ps < 0.0001 to 0.0192, respectively). Also, treatment with molnupiravir up to 12 h following infection with MERS-CoV significantly decreased both lung hemorrhage (day 6 post-infection: ps = 0.0004 to <0.0001), loss of body weight (days 2–6 post-infection: ps = 0.02 to <0.0001) and improved pulmonary function (day 3 post-infection: p < 0.0001 and p = 0.0002; day 4 post-infection: p < 0.0001 and p = 0.0008), whereas virus lung titer was decreased to the limit of detection on the 6th day of infection in all molnupiravir-treated groups [24].

Consequently, despite the potential *in vitro* antiviral activity of NHC against three known zoonotic coronaviruses and *in vivo* activity of molnupiravir against SARS-CoV and MERS-CoV, the grade of clinical benefits was associated with the time of starting of the drug following infection. Notably, considering the different kinetic of diseases in mice and humans, lung viral titers peak occurs earlier following SARS-CoV and MERS-CoV infection in mice compared with humans. Therefore, timely administration of molnupiravir during the first week of infection, which is concomitant with the peak of virus replication in humans, is clinically important. Although severe COVID-19 patients with long-lasting disease may benefit from even late administration of the drug [37–40]. It should be noticed that considering severe forms of zoonotic virus diseases in older patients, lack of drug efficacy evaluation in CoV-aged mouse models is an important limitation of the study [11]. Moreover, in this study, because of incompatibility between S glycoprotein and ACE2 receptors of mice, the efficacy of molnupiravir in *in vivo* condition was not evaluated against SARS-CoV-2 [41,42]. Three other studies evaluated the efficacy of molnupiravir against SARS-CoV-2 in the Syrian hamster, ferret, and mice implanted with human lung tissue models [28,43,44].

Rosenke *et al.* [28] carried out a series of studies to assess the effects of molnupiravir on SARS-CoV-2 replication in the Syrian hamster model. To evaluate *in vitro* inhibitory effect of NHC on SARS-CoV-2 replication, Calu-3 cells were pretreated with various concentrations of NHC. Results showed that treatment with NHC is associated with an 880-fold decrease in SARS-CoV-2 replication compared to control groups with an IC_50_ value of 414.6 nM. Following assessing *in vitro* effects of NHC, the efficacy of molnupiravir against SARS-CoV-2 was evaluated in the Syrian hamster model as a preclinical model of mild disease. One group of hamsters received a vehicle, while two treatment groups were treated with molnupiravir (250 mg/kg) 12 and 2 h before or 12 h after infection. Moreover, animals in the treatment groups received molnupiravir (250 mg/kg) every 12 h for 3 consecutive days.

The assessment of the oral cavity on days 2 and 4 after infection showed that levels of viral RNA (all ps ≥ 0.9999) and infectious titers (control vs pre-treatment; p = 0.5701; control vs post-treatment, p ≥ 0.9999) were similar between all the groups. The analysis of lung tissue samples on day 4 after infection revealed a significant decrease in lung tissue viral load of the pre-infection (p = 0.0091). Moreover, viral infectious titers in the lung tissues were significantly lower in the pre-infection (p = 0.0091) and post-infection (p = 0.0102) groups, compared to the control group.

The histopathological analyses of lung samples showed that lung lesions were less abundant in the treatment groups compared with the control group. Using immunoreactivity against SARS-COV-2 antigen to assess the lung samples showed that the control group had 4.71 and 3.68-times more signal than pre-infection and post-infection treatment groups, respectively. The results of the study showed the benefits of molnupiravir against SARS-CoV-2 in the animal model, supporting possible effects of the drug in the prevention of infection following high-risk exposure in healthy humans and treatment of patients with COVID-19 [28]. Considering the failure of remdesivir in the prevention of SARS-CoV-2 shedding from the upper respiratory tract in rhesus macaque model, this observation with molnupiravir would be a promising finding with a positive clinical application of molnupiravir in the management of COVID-19 patients [45].

In line with Rosenke *et al.* study, Cox *et al.* showed that molnupiravir not only could significantly decrease the viral load of the upper respiratory tract but also block the viral transition to untreated contact animals in the SARS-CoV-2-infected ferret model [28,43]. In the first phase of Cox *et al.* [43] study, it has been shown that NHC could inhibit SARS-CoV-2 in Vero E6 cells with half maximal effective concentration (EC_50_) and EC_90_ of 3.4 μM and 5.4 μM, respectively. Afterward, the efficacy of molnupiravir against SARS-CoV-2 was evaluated in the ferret model, which simulated the spread of the virus in the young human population since both transmit the virus with mild clinical manifestations. Four groups of ferrets were inoculated intranasally with the virus. Among them, three groups were treated with either 1% methylcellulose (as a vehicle) or 5 or 15 mg/kg molnupiravir, initiating 12 h after infection, whereas the fourth group received molnupiravir (15 mg/kg), starting 36 h after infection. In all the treatment groups, molnupiravir was administered twice a day for 4 days after infection. Nasal lavages were collected every 12 h, and blood samples were obtained every other day. Data analysis showed that administration of molnupiravir with both doses following 12 h of infection led to a statistically significant reduction of the shed virus load within 12 h. In addition, after 36 h of infection, administration of molnupiravir at the peak of virus shedding resulted in inhibition of the infectious particles release into nasal lavages within 36 h (ps < 0.001). Besides, SARS-CoV-2 RNA was significantly cleared in the nasal tissues in both 5 and 15 mg/kg^-1^ molnupiravir groups (ps = 0.0089 and 0.0081) compared with the control group. Finally, treatment with molnupiravir starting 12 h after the infection led to a significant drop in effector interferon-stimulated gene expression (p ≤ 0.044).

In the next phase of the study, co-housing infected ferrets with uninfected ferrets for 72 h showed that the virus transmission among these animals is rapid and efficient. To evaluate the efficacy of molnupiravir in the blocking of virus contact transmission, two groups of infected ferrets were treated with either molnupiravir (5 mg/kg, twice daily) or a vehicle, 12 h after infection (source ferrets), twice daily. Following 30 h of infection, the source ferrets were co-housed with uninfected and untreated ferrets for 3 days (contact ferrets). Afterward, the contact ferrets were monitored for 4 days. Data analysis showed that molnupiravir treated ferrets had significantly lower virus titers compared with the vehicle animals after 12 h of treatment initiation (p = 0.0003). Importantly, while the contact ferrets co-housed with vehicle-treated source ferrets initiated to shed the virus following 20 h of co-housing, no virus could be detected in the contact ferrets co-housed with molnupiravir treated source ferrets [43].

These data supported the beneficial effects of molnupiravir against SARS-CoV-2 infection in the preclinical models. Currently, the major clinical trials are conducting to assess the efficacy and safety of molnupiravir against SARS-CoV-2 [46,47]. To assess the proper antiviral doses of molnupiravir, Wahl *et al.* [44] carried out a study in immunodeficient mouse model implanted with human lung tissue which infected with the three known zoonotic coronaviruses and two endogenous SARS-like bat coronaviruses (WIV1-CoV and SHC014-CoV). Data of histopathological analysis showed that acute SARS-CoV-2 infection of these mice is highly cytopathic, which is similar to lung infection in humans. After inoculation of the human lung tissues of mice with SARS-CoV-2, high titers of virus particles were observed at 2, 6 and 14 days after exposure. Moreover, acute SARS-CoV-2 infection significantly is associated with gene pathways up-regulation, such as coagulation (p = 0.0453), regulation of cell death (p = 0.0030), acute inflammatory response (p = 0.0035), innate immune response (p = 0.0010), cytokine-mediated signaling (p = 0.0010), cytokine production (p = 0.0010), response to stress (p = 0.0010), inflammatory response (p = 0.0010), response to type-I interferon (p = 0.0011) and response to virus (p = 0.0010) pathways and leads to an effective and continuous innate immune responses in the lung tissues of mice. Furthermore, data analysis showed that administration of molnupiravir (500 mg/kg; twice daily) initiating 24 and 48 h after exposure to SARS-CoV-2 could inhibit SARS-CoV-2 titers (4.4 logs; p = 0.0002; 1.5 logs; p = 0.0019). Similarly, starting the treatment 12 h before infection significantly decreased virus titers by above 100,000-fold in two independent experiments (ps = 0.0002 and 0.0068). Finally, pathologic findings of SARS-CoV-2 infection were not observed in the mice treated with molnupiravir [44].

Previously, the clinical benefits of combination antiviral therapy have been shown in the treatment of viral infections such as the human immunodeficiency virus and influenza [48,49].

Abdelnabi *et al.* conducted a study to evaluate the effects of combination therapy of molnupiravir and favipiravir against SARS-CoV-2 [13]. to assess *in vivo* efficacy of molnupiravir against SARS-CoV-2 at the time of infection, SARS-CoV-2-infected hamsters were treated with either molnupiravir or a vehicle for 4 days. Results showed that molnupiravir therapy with doses of 75, 150, 200 and 500 mg kg^-1^, twice daily, led to 1.3 (p = 0.002), 1.9 (p < 0.0001), 3.3 (p < 0.0001) and 2.8 (p = 0.01) log10 reduction in the SARS-CoV-2 RNA copies/mg (lung tissue), respectively, demonstrating a dose-dependent effect of molnupiravir against SARS-CoV-2 in the hamster model. Similarly, molnupiravir therapy with doses of 150, 200 and 500 mg/kg, twice daily, was associated with 1.3 (p = 0.0002), 3.5 (p < 0.0001) and one (p = 0.0002) log10 reduction in the infectious virus titers; however, the dose of 75 mg/kg, twice daily did not reduce the infectious virus load. Notably, the doses of 75, 150 and 200 mg/kg significantly (ps = 0.0025, 0.005 and <0.0001) improved lung histopathology.

In the next phase of the study, hamsters were treated with either 200 or 500 mg/kg molnupiravir, twice daily, starting 24 h after infection. It has been shown that molnupiravir therapy with doses of 200 and 500 mg/kg twice daily led to 0.4 (p = 0.03) and 1 (p = 0.05, non-significant) log10 reduction in the SARS-CoV-2 RNA copies/mg (lung tissue), respectively. Furthermore, treatment with 500 mg/kg molnupiravir, twice daily, led to a modest (one log10) but significant (p = 0.0003) reduction in the infectious virus titers. Notably, treatment with 200 mg/kg molnupiravir twice daily led to a significant reduction in the histological lung disease score.

Finally, a combination of molnupiravir (150 mg/kg, twice daily) with favipiravir (300 mg/kg, twice daily, intraperitoneal injection) led to a significant reduction in the log10 RNA copies/mg (lung tissue) and histological lung pathology score compared with hamsters treated with favipiravir alone (both ps < 0.01). Moreover, the combination was associated with a significant reduction in infectious virus titers compared with groups treated with either molnupiravir (p < 0.05) or favipiravir (p < 0.001). Thus, all the studied doses of molnupiravir alone or in combination with favipiravir seem to be safe with no obvious adverse effects [13]. The published pre-clinical studies investigating the antiviral properties and safety profile of NHC and molnupiravir against SARS-CoV-2 are summarized in Table 1.

**Table 1.** Pre-clinical studies investigating the antiviral properties and safety of β-d-N 4-Hydroxycytidine and molnupiravir against SARS-CoV-2.

Study design		*In vitro* dose (NHC)	Animal dose (molnupiravir)	Outcomes		Study, year, and country
*In vitro*	*In vivo*			Efficacy	Safety	
Vero E6 cells, Calu-3 cells	Mice	1–10 μM for 48 h	50, 150, and 500 mg/kg twice daily	Significant dose-dependent reduction in the lung virus titer	CC50 >10 μM, no toxicity at doses up to 100 μM	Sheahan *et al.*, 2020, USA
Calu-3 cells	Syrian hamster	IC_50_ 0.14 μM	250 mg/kg 12 and 2 h before or 12 h after infection; or every 12 h for 3 consecutive days	Significant decrease in lung tissue viral load	Minimal cellular toxicity up to 40 μM	Rosenke *et al.*, 2020, USA
Vero E6 cells	Ferrets	EC_50_ μM	5 or 15 mg/kg twice daily 12 h after infection or 15 mg/kg twice daily 36 h after infection	Significant decrease of viral load in nasal tissues	No phenotypically overt adverse effects	Cox *et al.*, 2021, USA
	Mice		500 mg/kg, twice daily 24 and 48 h after exposure	Significant decrease in lung tissue viral load		Wahl *et al.*, 2021, USA
	Syrian gold hamsters		75, 150, 200, and 500 mg/kg twice daily for 4 days	Significant decrease in lung tissue viral load except for 75 mg/kg	No obvious adverse effects	Abdelnabi *et al.*, 2021, Belgium
	Syrian gold hamsters		Molnupiravir (150 mg/kg, twice daily) with favipiravir (300 mg/kg, twice daily)	Significant decrease in lung tissue viral load	No obvious adverse effects	Abdelnabi *et al.*, 2021, Belgium
Mammalian cells		EC_50_ of 0.3 μM		Inhibits viral replication	Dose-dependent (up to 3 μM) mutagenic effect	Zhou *et al.*, 2021, USA

NHC: β-d-N4-hydroxycytidine; μM: Micrometer; CC_50_: Half-maximal cytotoxic concentration; EC_50_: Half-maximal effective concentrations; IC_50_: Half-maximal inhibitory concentration.

#### Cytotoxicity & safety profile

Several studies evaluated the cytotoxicity profile of NHC cell culture models and showed favorable cytotoxicity profile [29–35]. In Painter *et al.* study, NHC showed good tolerability after 7-day repeated dosing (up to 1000 mg/kg/day) [36].

Furthermore, Sheahan *et al.* study showed that NHC had no cytotoxicity in the Calu-3 cells cultures with half-maximal cytotoxic concentration (CC_50_) >10 μM. Moreover, in primary human airway epithelial cell cultures, NHC showed no toxicity at doses up to 100 μM [28]. Besides, Rosenk *et al.* [28] evaluated the cellular toxicity of NHC in Calu-3 cells and indicated minimal cellular toxicity at the highest NHC concentration used for the analysis of SARS-CoV-2 replication (40 μM). Also, in Abdelnabi *et al.* study, all the studied doses of molnupiravir alone or in combination with favipiravir seem to be safe with no obvious adverse effects [13]. Besides, according to Cox *et al.* study in the ferret model, the use of molnupiravir was associated with no phenotypically overt adverse effect [43].

Zhoe *et al.*, in opposition to the previous literature reports, showed that although NHC could inhibit SARS-CoV-2 through lethal mutagenesis, it may have mutagenic effect on mammalian cells. Expression of *HPRT* gene in CHO-K1 cells could lead to sensitivity to the toxic base analog 6-thioguanine. Consequently, inactivation of the HPRT gene/protein function is correlated with resistance to 6-thioguanine. Zhoe and colleagues used loss of HPRT gene function in CHO-K1 cells to evaluate for DNA mutagenesis activity of ribavirin, favipiravir and ribonucleoside analog NHC. Result showed that ribonucleoside analog NHC had mutagenic effects on the cells in a dose-dependent manner (up to 3 μM), while favipiravir exhibited undetectable mutagenic activity up to a concentration of 300 μM. Likewise, ribavirin exhibited mutagenic properties at 300-fold the concentration of the similar ribonucleoside analog NHC mutagenic properties. These findings highlighted the concerns regarding mutagenic effects of ribonucleoside analog NHC on human DNA and following risk of cancer development, potential harm to fetus, precursors of egg and sperm and teratogenic effects. Consequently, not only molnupiravir should not be used during pregnancy, but also using contraceptive methods in male and female should not be ignored [50].

## Pharmacokinetics

Owning to poor human bioavailability of NHC, the 5′-isopropylester of NHC, EIDD-2801, with increased oral bioavailability was synthesized. EIDD-2801 is immediately converted to the active antiviral NHC by plasma esterase. Given the fact that SARS-CoV-2 is mainly involved in the respiratory system, levels of drugs in lung tissue seem to be the best indicator of therapeutic potential. The NHC lung concentrations were detectable in both pre-treatment (18.80 ± 5.97 nmol/gr _lung_) and post-treatment (17.56 ± 5.49 nmol/gr _lung_) groups. Estimated concentrations of molnupiravir in the lungs of animals were 15.04 ± 4.78 μM and 14.05 ± 4.39 μM in the pre-infection and post-infection groups, respectively. Since molnupiravir is rapidly hydrolyzed to NHC following absorption, molnupiravir was not detected in the lung tissue [10,23].

A phase I, randomized, double-blind, placebo-controlled ascending-dose trial was conducted on 64 and 56 healthy individuals to evaluate the human safety, tolerability and pharmacokinetics of single and multiple oral doses of molnupiravir [23]. In the single-dose part of the trial, individuals were given a single dose of 50–1600 mg/dose molnupiravir (50–800 mg oral solution and 1600 mg capsule), while individuals in the multiple-dose part received capsule formulation with doses of 50–800 mg/dose, twice daily, for 5.5 days. All cases were followed up for 2 weeks after treatment. Furthermore, a randomized, open-label, crossover assessment of the high-fat food effects on the pharmacokinetics of molnupiravir capsule formulation was carried out on ten healthy individuals. The mean ages of participants were 39.6, 36.5 and 45.3 in the single-dose, multiple-dose and food-affected evaluation parts of the trial, respectively.

Molnupiravir concentrations were mostly lower than the limit of quantification at single doses of ≤800 mg/dose, but quantifiable in at least one time-point between 0.25 and 1.5 h following administration of higher doses of molnupiravir (1200 and 1600 mg/dose) in all individuals. Evaluation of single-dose pharmacokinetics of molnupiravir (≥600 mg/dose) showed that the maximum plasma concentration (Cmax) and time to Cmax (Tmax) values were up to 13.2 ng/ml and between 0.25 and 0.75 h, respectively. Furthermore, after molnupiravir administration, NHC appeared promptly in plasma after administration of lower doses of molnupiravir (≤800 mg/dose), with a median Tmax of 1.00 h and eliminated from plasma in an essentially monophasic manner, with geometric mean terminal elimination half-lives of 0.910–1.29 h. However, a slight delay in Tmax (1.75 and 1.50 h) and longer mean terminal elimination half-life (1.81 and 4.59 h) values were observed with the increase of dose (1200 and 1600 mg). The evaluation of plasma concentration–time profiles showed that the mean Cmax and area under the concentration–time curve from time zero extrapolated to infinity values increased in a dose-proportional manner. Furthermore, at doses between 50 mg and 1600 mg/dose, 0.820–6.70% of the dose was excreted in urine as NHC form.

In general, concentrations of molnupiravir were below the limit of quantification at multiple-dose of ≤400 mg/dose of the drug. Following multiple-dose administration of molnupiravir, NHC appeared quickly in plasma on both days 1 and 6 with Tmax of 1.00–1.75 h, respectively. Plasma concentrations of NHC dropped in a monophasic manner for all cases on day 1, and for most of the individuals who received lower doses of molnupiravir (≤400 mg/day) on day 6, with mean terminal elimination half-lives of 0.918–1.18 h; however, on day 6, biphasic elimination was observed for those received higher doses of the drug (≥600 mg/day), with a mean terminal elimination half-life of 7.08 h (800 mg/day).

The mean accumulation ratios based on area under the plasma concentration–time curve during a dosing interval (0.938 and 1.16) and Cmax (0.843 and 1.10) values showed no evidence of accumulation across all dose levels. On days 1 and 6, both mean Cmax and area under the plasma concentration–time curve from time zero extrapolated to infinity increased in a dose-proportional manner. Moreover, on both days 1 and 6, 0.854–3.61% of the dose was excreted in urine as NHC. Finally, it has been shown that the rate of absorption is slightly faster for the solution form of the drug than capsule formulation; however, the extent of absorption is similar for both formulations [23].

Results of food affected evaluation part of the study revealed that administration of molnupiravir capsule in the fed states results in a median delayed Tmax of 3 h compared with 1 h in the fast states. Furthermore, although mean Cmax was higher in the fasted state than the fed state, mean area under the plasma concentration–time curve from time zero extrapolated to infinity was similar between both states, reflecting that regardless of the rate, the extent of absorption was higher in the fasted states [23]. Notably, the pharmacokinetics of drugs may be changed in COVID-19 patients, especially in severe cases [51,52]. The well-known effects of early antiviral agents in patients with milder clinical manifestations shows the importance of performing studies evaluating the pharmacokinetics of molnupiravir in outpatients with mild to moderate COVID-19. Khoo *et al.* [53] carried out a phase I dose-escalation, open-label, randomized, controlled Bayesian adaptive trial to assess pharmacokinetic and safety of molnupiravir in mild to moderate patients with COVID-19 within 5 days of symptom onset. A total of 18 patients were randomized 1:2 to receive standard care alone or in combination with molnupiravir (300, 600 or 800 mg), every 12 h for 5 days. The mean (range) age of patients was 56 (22–80), and the majority of them were female (72.7%). At the end of the trial, 75%, 100% and 75% of the patients received full treatment in the doses of 300, 600 and 800 mg, respectively. In line with the trial in healthy adults, EIDD-2801 was only detected at low concentrations within 1 h after starting the treatment. Geometric mean (coefficient of variation [CV]) area under the time-concentration curve (0–4 h) of NHC following single-dose administration at day 1 were 3210 (40.5), 4610 (33.7) and 9240 (41.0) ng.h ml^-^^1^, in the doses of 300, 600 and 800 mg, respectively, with corresponding Cmax of 1490 (29.4), 2230(38.2) and 4440 (45.2) ng ml^-^^1^ and median T max of 1.5, 1.5 and 2 h. Following multiple-dose administration of molnupiravir at day 5, assessment of geometric mean (CV) area under the time-concentration curve (0–4 h) (ng ml^-^^1^), C max (ng ml^-^^1^) and Tmax (h) of NHC in 300 mg (3470 [42.4], 1620 [51.0] and 1), 600 (3880 [56.3], 1820 [84.6] and 1), and 800 mg (7880 [39.0], 4180 [28.1], and 2) doses showed no accumulation between days 1–5 [53].

## Clinical trials

### Safety & efficacy in clinical trials

Our knowledge on molnupiravir safety profile comes from both pre-clinical studies and randomized controlled trials in healthy individuals and COVID-19 patients [23,24,28,43,44,53].

In Painter *et al.* trial [23], results of the single-dose part of the trial showed that 35.4% and 43.8% of individuals reported adverse events after administration of molnupiravir and placebo, respectively. No severe adverse events were observed in both groups. Moderate headache and vomiting were reported in one case in the patients treated with 400 mg/dose molnupiravir and placebo, respectively. The most common reported adverse event in the individuals received molnupiravir (12.5%) and placebo (18.8%) was headache. Similarly, in the multiple-dose part of the study, the rate of adverse events was lower in molnupiravir-treated compared with placebo-administrated cases (42.9% vs 50.0%). All reported adverse events were mild, with the exception of one case with moderate influenza-like syndrome in the cases treated with 200 mg/dose molnupiravir. The most common reported adverse event was diarrhea, which was reported by 7.1% of individuals in each group of intervention and control. Only one case was excluded from the study due to mild, truncal, maculopapular, pruritic rash on the fourth day after administration of 800 mg/dose molnupiravir. Also, in the food-affected assessment part of the study, one mild adverse event was reported by three cases. Finally, no clinically significant changes or dose-related trends were reported in vital signs, and laboratory and electrocardiogram data. Importantly, due to the fact that plasma exposures were expected to be efficacious according to animal models of influenza, dose escalations were discontinued before reaching a maximum tolerated dose [23,25].

In Khoo *et al.* [53] trial, no serious adverse events were reported, and only mild adverse events were reported in all the patients who received 300 and 600 mg molnupiravir, while in 25% and 83% of patients in the 800 mg and the control patients were observed, respectively. The most common symptoms were loss of smell or taste, diarrhea, nausea, cough and flu-like symptoms, which are consistent with mild to moderate manifestation of the disease. According to the results of the phase I trial, a dose of 800 mg every 12 h for 5 days has been recommended for a blinded placebo-controlled randomized phase II trial to evaluate the efficacy of molnupiravir in patients with COVID-19. These results of these trials revealed a well-tolerated safety profile of molnupiravir; however, the results of the ongoing trial may further bring more clarity. Bernal *et al.* carried out a phase III, double-blind, randomized, placebo-controlled trial to assess the efficacy and safety of early administration of molnupiravir (800 mg, twice daily for 5 days) within 5 days following clinical presentation in 1433 outpatients with mild-to-moderate COVID1-19 and at least one risk factor for progression to severe disease [54]. Data analysis showed that the risk of hospitalization or mortality was significantly higher in the control group compared with the intervention group (14.1% vs 7.3%; p = 0.001). There was no significant difference regarding the reported adverse events in the placebo and intervention groups (33.0% vs 30.4%, respectively). Finally, nine deaths were reported in the control group and one death in the intervention group.

### Ongoing clinical trials

We searched clinicaltrials.gov from database inception to 23 July 2021, to find the ongoing clinical trials of molnupiravir in healthy individuals or patients with COVID-19. At the time of writing the manuscript, six ongoing clinical trials are being carried out to evaluate the safety and efficacy of molnupiravir in COVID-19 patients (Table 2). Among them, two studies are based in the USA, one in the UK and South Africa, and two are in multiple countries. The locations of one study have not been determined. The sample size of the studies ranges from 96 to 1450, with a cumulative sample size of 3986. The clinical status of COVID-19 varies from outpatients with mild disease to hospitalized cases with severe disease. In one study, the preventive effect of molnupiravir was evaluated in individuals residing with COVID-19 patients. The primary endpoints of the studies include hospitalization, mortality and adverse events rate, discontinuation due to an adverse event, percentage of participants with COVID-19, viral clearance, recovery time, and determination of appropriate dose for efficacy evaluation. In all the trials, the route of administration is oral, and a placebo has been used. The dose of molnupiravir ranges from 200 to 800 mg every 12 h for 5 days. A planned interim data analysis of two ongoing clinical trials in outpatients and hospitalized patients with COVID-19 showed that molnupiravir could inhibit SARS-CoV-2 replication in both settings. Molnupiravir administration is associated with a lower hospitalization and mortality rate in the outpatient, while it is unlikely to have beneficial effects in hospitalized patients with COVID-19. Moreover, the beneficial effects of the drug seem to be higher with the 800 mg every 12 h dose, in patients enrolled within 5 days of symptoms onset, and among individuals with risk factors for poor prognosis COVID-19, such as diabetes mellitus and obesity. Consequently, according to the results of the interim analysis of data, the investigators have decided to discontinue the trial of hospitalized patients and evaluate the effects of 800 mg (twice daily, for 5 days) molnupiravir in outpatients with COVID-19 [46,47]. Furthermore, in line with the results of preclinical and clinical studies, molnupiravir showed a good safety profile in both ongoing trials; accordingly, neither death nor serious adverse events were related to the drug were detected to date. A phase III, comparative, randomized, multicenter clinical trial is conducting to evaluate the safety and efficacy of molnupiravir (800 mg twice daily for 5 days) in 1218 patients with mild COVID-19. According to the interim results of 741 individuals, the administration of molnupiravir could decrease hospital admission rate (1.89% vs 6.22%; p = 0.0027) over 14 days and median time to clinical improvement (8 days vs 12 days; p = 0.0001). Also, the percentage of individuals with a 2-point decline in WHO Clinical Progression Scale was higher in the molnupiravir group compared with the control group (days 5 [63.43% vs 22.33%; p ≤ 0.0001], 10 [78.96% vs 49.49%; p ≤ 0.0001] and 14 [81.55% vs 73.22%; p = 0.0150]). The amount of patients with negative SARS-CoV-2 reverse transcription-polymerase chain reaction (RT-PCR) negative test was higher in the molnupiravir group than the control group (days 5 [77.35% vs 26.07%; p ≤ 0.0001], 10 [94.03% vs 57.20%; p ≤ 0.0001] and day 14 [97.01% vs 85.21%; p ≤ 0.0001]). Finally, nausea, diarrhea, and headache were the most common reported adverse events. All observed adverse events were mild and did not lead to drug discontinuation. Importantly, given the observed genotoxic effects of molnupiravir in the *in vitro* studies, evaluating the effects of this medicine should be conducted only in selected patients most likely to benefit along with considering its genotoxic and teratogenic effects [23,24,28,43,44,46,47,50,53].

**Table 2.** Ongoing clinical trials investigating the therapeutic effects of molnupiravir for the treatment of COVID-19.

ID	Status	Design	Country	Population (n)	Intervention group(s)	Comparison group(s)	Primary outcomes
NCT04575597	Recruiting	phase II/III, randomized, placebo-controlled, double-blind clinical trial	Multicounty	Non-hospitalized adults with COVID-19 (1450)	Molnupiravir every 12 h for 5 days (200, 400 or 800 mg)	Placebo every 12 h for 5 days	Hospitalization Mortality Adverse events discontinuation due to an adverse event
NCT04575584	Terminated	phase II/III, randomized, placebo-controlled, double-blind clinical trial	Multicounty	Hospitalized adults with COVID-19 (304)	Molnupiravir every 12 h for 5 days (200, 400 or 800 mg)	Placebo every 12 h for 5 days	Time to recovery Adverse events discontinue due to an adverse event
NCT04405739	Recruiting	phase IIa randomized, placebo-controlled, double-blinded clinical trial	USA	Newly hospitalized adults with COVID-19 (96)	Molnupiravir (six doses), every 12 h for 5 days	Placebo every 12 h for 5 days	Viral clearance adverse events Serious adverse events
NCT04405570	Completed	phase IIa randomized, double-blind, placebo-controlled trial	USA	Symptomatic adult outpatients with COVID-19 (204)	Molnupiravir (nine doses), every 12 h for 5 days	Placebo every 12 h for 5 days	Viral clearance Adverse events
NCT04939428	Recruiting	phase III, multicenter, randomized, double-blind, placebo-controlled trial	Not Provided	Adults who reside with a person with COVID-19 (1332)	Molnupiravir every 12 h for 5 days (200 mg)	Placebo every 12 h for 5 days	Percentage of participants with COVID-19 Adverse events discontinuation
NCT04746183	Recruiting	phase I: Open-Label randomized controlled trial	UK South Africa	Adults with COVID-19 (600)	Molnupiravir (300, 600, 800 mg) every 12 h for 5 days	Placebo every 12 h for 5 days	Determination of a dose(s) for efficacy evaluation
		phase II: Blinded controlled parallel trial			Molnupiravir 800 mg, every 12 h for 5 days		Determination of activity and safety

## Limitations

The present comprehensive review aimed to gather all the available evidence regarding pharmacokinetics, pharmacodynamics, and clinical trials of molnupiravir in the management of COVID-19; however, most of the clinical trials evaluating the efficacy of the medicine have not been published yet. Furthermore, a few numbers of referenced studies in the manuscript are in the preprint status. Consequently, caution should be taken regarding these until publication.

## Conclusion & future perspective

Despite the considerable progress in understanding the pathophysiology of COVID-19, the well-documented efficacy of some therapeutic approaches and the development of efficacious vaccines, the number of new cases of COVID-19 is soaring. Unvaccinated populations and emerging newer variants seem to be the main causes for this situation; which requires developing new vaccines, increase of vaccination rates, booster doses for high-risk vaccinated populations as well as developing new antiviral agents. It should be kept in mind that similar to some other respiratory infections, vaccination alone may not be a guarantee for COVID-19 prevention, which necessitates new antivirals for the management of COVID-19. Directions for future research have been proposed with the aim of repurposing and developing antiviral drugs for the prevention and treatment of COVID-19 [2–5]. Currently, a series of potential investigational antivirals, such as nitric oxide (NO), plitidepsin, AT-527, Paxlovid, and molnupiravir have been shown beneficial effects in the management of COVID-19. NO is an inexpensive antiviral agent with the special capability to cause pulmonary vasodilation. Besides, NO could reduce oxygen requirement and progression of the disease in patients with COVID-19. Similar to other viruses, risk of antiviral resistance is a clinically significant problem in the management of the disease. Plitidepsin could prevent viral resistance by affecting host proteins. Based on pre-clinical investigations, this antiviral is about 27-times more potent than remdesivir. AT-527 is another oral RdRp inhibitor that is in phase III clinical trials of COVID-19. Paxlovid (PF-07321332; ritonavir) and PF-07304814 are two promising oral and parenteral antivirals developed by Pfizer pharmaceutical company to combat the pandemic. On December 22, 2021, the FDA approved the first oral antiviral Paxlovid for the treatment of mild to moderate COVID-19 in patients aged more than 12 years weighing over 40 kg under an emergency use authorization (EUA). Paxlovid is a combination ritonavir and a novel protease inhibitor PF-07321332 that blocks a protease needed for the replication process. Afterwards, the FDA approved the use of molnupiravir for mild to moderate COVID-19 only for patients aged 18 years and over due to its probable effects on bone and cartilage growth [54–60].

Oral antivirals can be used easily in earlier stages of infection in selected outpatients. Currently, it seems that Paxlovid and molnupiravir are two most promising oral antiviral against COVID-19. Drug availability, administration in health care settings, cost, parenteral route of administration, and possible reduced susceptibility of Omicron variant due to numerous mutations in the spike protein may be considered as disadvantages of anti-SARS-CoV-2 monoclonal antibodies. Oral antiviral medications could be administrated at the first clinical manifestations of the disease or following exposure to the virus. This is big advantage of molnupiravir and Paxlovid over parenteral antiviral drugs and neutralizing antibodies. Notably, molnupiravir is a less complicated product compared with Paxlovid making it the most widely available oral antiviral medication in the management of COVID-19 [54–60].

In the present review, we aimed to discuss the current literature on the pharmacodynamics, pharmacokinetics, and clinical trials of molnupiravir in the management of COVID-19. Preclinical studies have shown the antiviral properties of NHC against SARS-CoV-2, while the *in vitro* cytotoxicity profile studies showed controversial results. As far as available clinical trials results, molnupiravir seems to be safe at doses up to 800 mg per dose (every 12 h for 5 days) in both healthy individuals and COVID-19 patients; however, given its probable genotoxic effects, further well-designed randomized clinical trials with large sample size and longer follow-up periods are still required to confirm the efficacy and safety of molnupiravir in patients with COVID-19. Finally, in future clinical trials of molnupiravir, monitoring of the long-term adverse events, probable effects on bone and cartilage growth, and possible genotoxic effects along with its potential effectiveness in patients with COVID-19 should be considered.

**Executive summary**

**Background**

- Despite the increase in the number of vaccinated people and the considerable effects of COVID-19 vaccines in protecting against severe illness, the pandemic is not yet under control.
- Detecting new variants of SARS-CoV-2 and reporting patients with severe illness has highlighted the importance of identifying effective and safe medications against COVID-19.
- Molnupiravir is a small-molecule prodrug with promising antiviral properties against SARS-CoV-2.

**Virology & pathogenesis**

- Given the important role of RdRp in the replication and transcription process of SARS-CoV-2 RNA, it has been a key target in the ongoing search for novel antiviral medications with acceptable efficacy and safety profile against COVID-19.

**Molnupiravir mechanism of action**

- Molnupiravir is a well-known viral RdRp inhibitor with remarkable mutagenic effects on viral RNA.

**Molnupiravir efficacy in pre-clinical studies**

- NHC (the active form of molnupiravir) is a potent antiviral against SARS-CoV-2 in infected cell culture models.
- Molnupiravir has shown antiviral activity against SARS-CoV-2 through reducing viral load in the respiratory tract and virus transmission in animal models.

**Molnupiravir safety in pre-clinical studies**

- There are controversial data regarding the *in vitro* cytotoxicity profile of NHC. While most of the preclinical studies have demonstrated a favorable cytotoxicity profile, possible genotoxic effects should not be ignored.
- No significant short-term adverse effect has been reported in animal models following the administration of molnupiravir.

**Molnupiravir in clinical trials**

- According to available clinical trials, the antiviral medication seems to be safe at a dose of 800 mg, twice daily for 5 days, in both healthy and COVID-19 populations.
- Early treatment with molnupiravir (800 mg, twice daily for 5 days) could decrease the risk of hospitalization or mortality in mild-to-moderate COVID-19 patients with one or more risk factors for progression to severe forms of the disease.
- Conducting future randomized clinical trials are still needed to evaluate the long-term safety profile of molnupiravir as well as its efficacy in both vaccinated and unvaccinated populations.
